# Supplementary material for: Results of a Clinical Trial Showing Changes to the Faecal Microbiome in Racing Thoroughbreds after Feeding a Nutritional Supplement
Source: Vet Sci. 2022 Dec 30;10(1):27. doi: 10.3390/vetsci10010027 (PMC9861731; doi:10.3390/vetsci10010027)
Supplement: Supplementary file 1 [file vetsci-10-00027-s001.zip › vetsci-2017258-supplementary.pdf]

Supplemental Table S1. List of 22 horses with sex, date of birth, age at start of study, sampling information, medications, racing history and outcomes.

Horse No.	Group	Sex†	Date of Birth	Age at Start	No. of Samples	Sample Group	Medications‡	1st Sample	Medications‡
106	Control	c	04 February 2017	2.91	3	1		02 January 2020	
108	Control	c	04 February 2017	2.91	3	1		02 January 2020	
110	Control	c	19 April 2017	2.70	3	1		02 January 2020	
114	Control	f	09 March 2017	2.82	3	1		02 January 2020	
122	Control	c	12 April 2017	2.72	3	1		02 January 2020	
123	Control	f	04 March 2017	2.83	3	1		02 January 2020	
103	Control	g	22 January 2016	4.06	3	2	2 Jan Omep	13 February 2020	
105	Control	f	30 April 2017	2.79	3	2		13 February 2020	
133	Control	c	20 April 2017	2.82	3	2		13 February 2020	
134	Control	c g	22 April 2017	2.81	3	2		13 February 2020	
107	Treatment§	c	03 May 2017	2.67	3	1		02 January 2020	
129	Treatment§	c g	11 February 2017	2.89	3	1	20 Jan gelded, PBZ*	02 January 2020	
117	Treatment	c	07 April 2017	2.74	3	1		02 January 2020	
119	Treatment	f	27 January 2017	3.04	3	1		13 February 2020	
120	Treatment	f	06 May 2017	2.77	3	1	15, 30 Jan PBZ	13 February 2020	9 Mar PBZ
126	Treatment	c	06 February 2017	2.90	3	1		02 January 2020	
127	Treatment	c	26 March 2017	2.77	3	1		02 January 2020	28 Jan PBZ, 29 Jan Omep
101	Treatment	f	25 February 2017	2.97	2	2		13 February 2020	
102	Treatment	f	21 January 2017	3.06	3	2		13 February 2020	
109	Treatment	c	22 January 2017	2.94	3	2		02 January 2020	
115	Treatment	f	25 February 2017	2.97	3	2		13 February 2020	13 Mar PBZ
124	Treatment	c	06 April 2017	2.74	3	2		02 January 2020	

*	also given methylprednisilone acetate injection (Depo-Medrone™ V)
†	c = colt; c g = colt and was then gelded; f = filly
‡	Omep = Omeprazole (GastroGard); PBZ = phenylbutazone (); date(s) raced prior to sampling
§	Included in the intention to treat analysis

Supplemental Table S1. List of 22 horses with sex, date of birth, age at start of study, sampling information, medications, racing history and outcomes continued.

Horse No.	Group	2nd Sample	Medications‡	3rd Sample	Racing history	No. of Races	Last race prior to trial	Improvement Lenient	Improvement Stringent
106	Control	13 February 2020		26 March 2020	2019	1	25 October 2019	No	No
108	Control	13 February 2020		26 March 2020	2019	4	12 October 2019	No	No
110	Control	13 February 2020		26 March 2020	2019	6	01 November 2019	Yes	No
114	Control	13 February 2020		26 March 2020				No	No
122	Control	13 February 2020		26 March 2020				No	No
123	Control	13 February 2020		26 March 2020				Yes	No
103	Control	26 March 2020	5 May PBZ	15 May 2020	2018-19	7	06 July 2019	No	No
105	Control	26 March 2020		15 May 2020				No	No
133	Control	26 March 2020	23 Apr, 6 May PBZ	15 May 2020	2019	1	05 October 2019	No	No
134	Control	26 March 2020	2 Apr gelded PBZ*	15 May 2020	2019	2	28 December 2019	No	No
107	Treatment§	13 February 2020		26 March 2020				Yes	No
129	Treatment§	13 February 2020		26 March 2020	2019	3	23 November 2019	No	No
117	Treatment	13 February 2020		26 March 2020				Yes	Yes
119	Treatment	26 March 2020		15 May 2020	2019	1	02 November 2019	No	No
120	Treatment	26 March 2020	5 May PBZ	15 May 2020				Yes	Yes
126	Treatment	13 February 2020		26 March 2020				Yes	Yes
127	Treatment	13 February 2020		26 March 2020				Yes	Yes
101	Treatment	26 March 2020		15 May 2020	2019	2	06 September 2019	Yes	Yes
102	Treatment	26 March 2020	9 Apr. PBZ	15 May 2020				Yes	Yes
109	Treatment	13 February 2020		26 March 2020				Yes	Yes
115	Treatment	26 March 2020	21 Apr PBZ	15 May 2020	2020	4	3, 29 Jan, 15 Feb, 12 Mar 2020	No	No

124	Treatment	13 February 2020	23 Mar PBZ	26 March 2020		Yes	Yes

* also given methylprednisilone acetate injection (Depo-Medrone™ V)

† c = colt; c g = colt and was then gelded; f = filly

‡ Omep = Omeprazole (GastroGard); PBZ = phenylbutazone (); date(s) raced prior to sampling

§ Included in the intention to treat analysis

Supplemental Table S**2**. Relative bacterial abundances reported in previously published studies.[1,3,8]

Ref. No.	Horse	F:B	Firmicutes (F)	Bacteroidetes (B)	Proteobacteria
8	1†	5.7	74	13	2
	2†	1.7	52	31	4
	Mean (SD)	3.7 (2.8)	63 (16)	22 (13)	3.0 (1.4)
	3‡	1.7	47	28	11
	4‡	3	57	19	2
	Mean (SD)	2.4 (0.9)	52 (7)	24 (6)	6.5 (6.4)
	5§	3.3	56	17	3
	6§	1.9	48	26	1
	Mean (SD)	2.6 (1.0)	52 (6)	22 (6)	2.0 (1.4)

Ref. No.	Healthy	F:B	*Firmicutes (F)*	*Bacteroidetes (B)*	*Proteobacteria*
1	1	3.24	39.5	12.2	42.7
	2	5.41	48.7	9.0	36.5
	3	8.76	78.0	8.9	3.4
	4	3.45	73.4	21.3	–
	5	5.30	73.1	13.8	–
	6	3.61	72.6	20.1	–
	Mean (SD)	4.80 (2.09)	68.1 (16.0)	14.2 (5.4)	10.1 (21.1)

Ref. No.	Colitis	F:B	*Firmicutes (F)*	*Bacteroidetes (B)*	*Proteobacteria*
	1	1.07	49.2	46.0	3.1
	2	2.53	65.4	25.8	7.1
	3	1.35	51.9	38.5	3.0
	4	0.85	37.5	44.2	6.1
	5	0.65	33.1	50.7	7.5
	6	0.31	11.9	38.3	48.5
	7	1.23	33.5	27.3	2.4
	8	0.81	38.8	48.0	5.0
	9	0.41	28.3	69.5	–
	10	6.95	39.6	5.7	52.5
	Mean (SD)	0.76	30.3 (14.5)	40.0 (17.1)	18.74 (20.2)

Ref. No.		F:B	Firmicutes (F)	Bacteroidetes (B)	Proteobacteria
3	Mean¶	11.98	43.7	3.7	3.8

† starch-fed with haylage to represent performance horses in active training

‡ forage/grass fed to represent racehorses at rest

§ haylage fed to represent racehorses at rest

¶ 4 samples from 2 horses

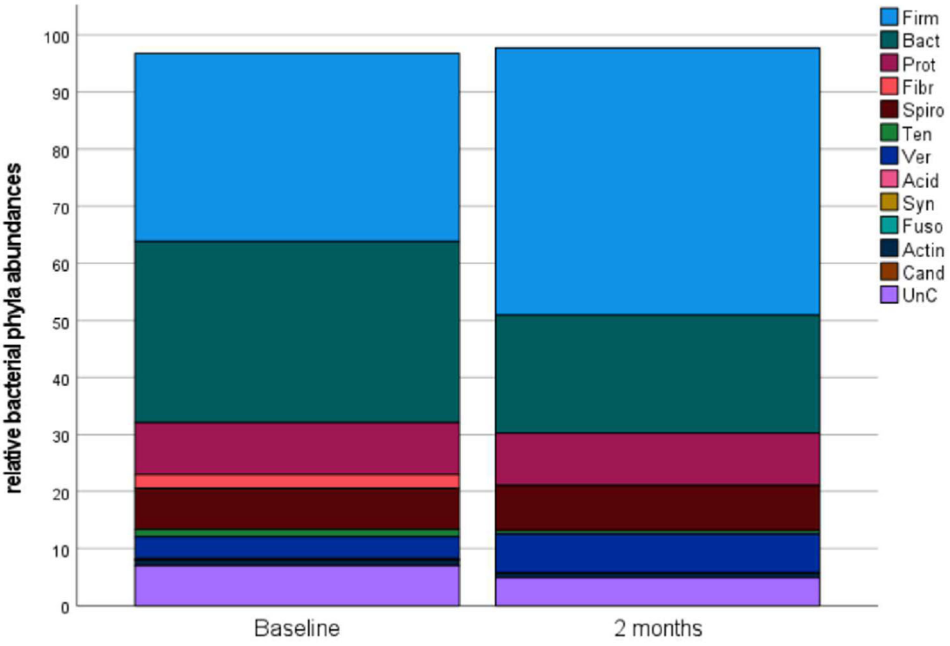

Figure S1. Stacked bar charts showing mean relative bacterial phyla abundances for 4 horses at baseline and after 2 months of supplementation in the pilot study.

A. Baseline samples

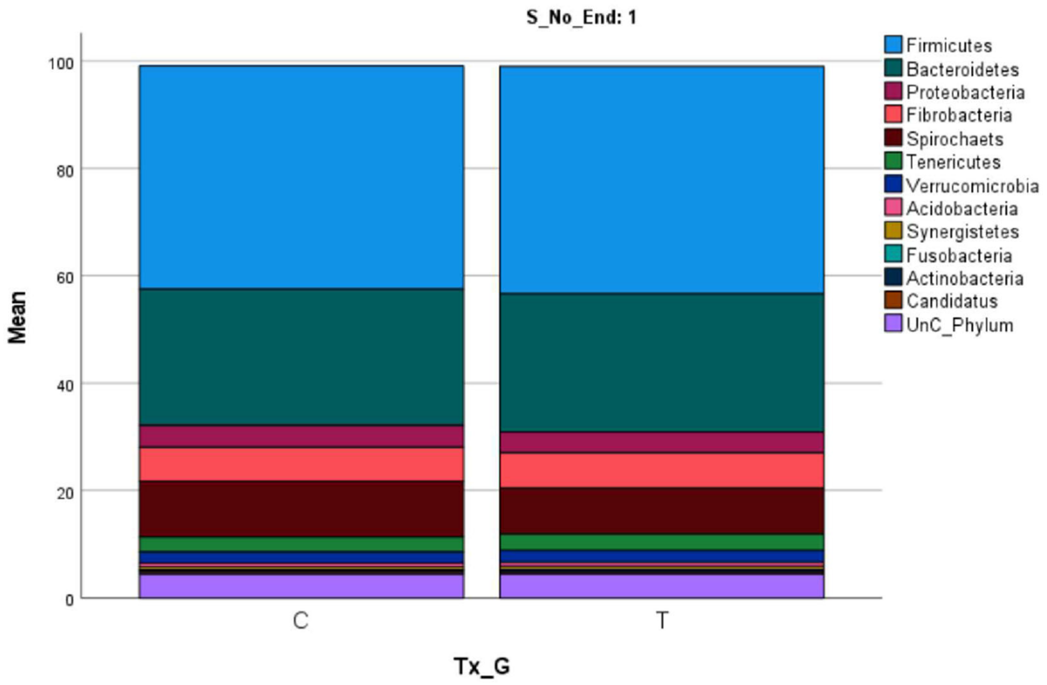

B. 12 week samples

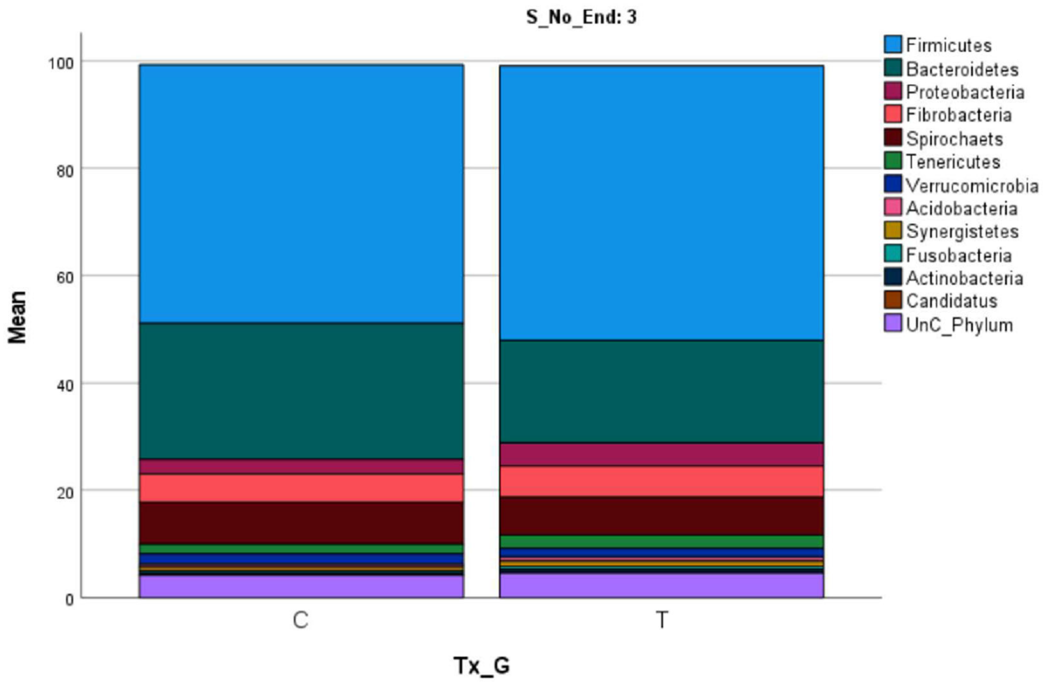

Figure S2. Stacked bar charts showing relative phyla abundances for all control (C) and treated (T) horses at: A. Baseline and B. 12 weeks.

101: improvement

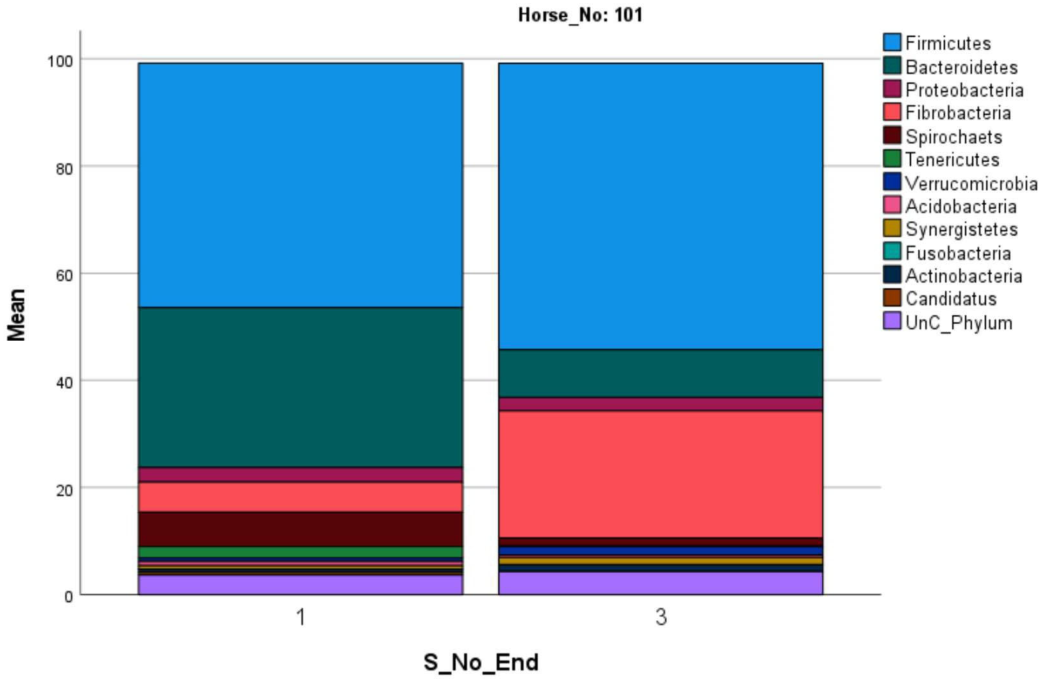

102: improvement

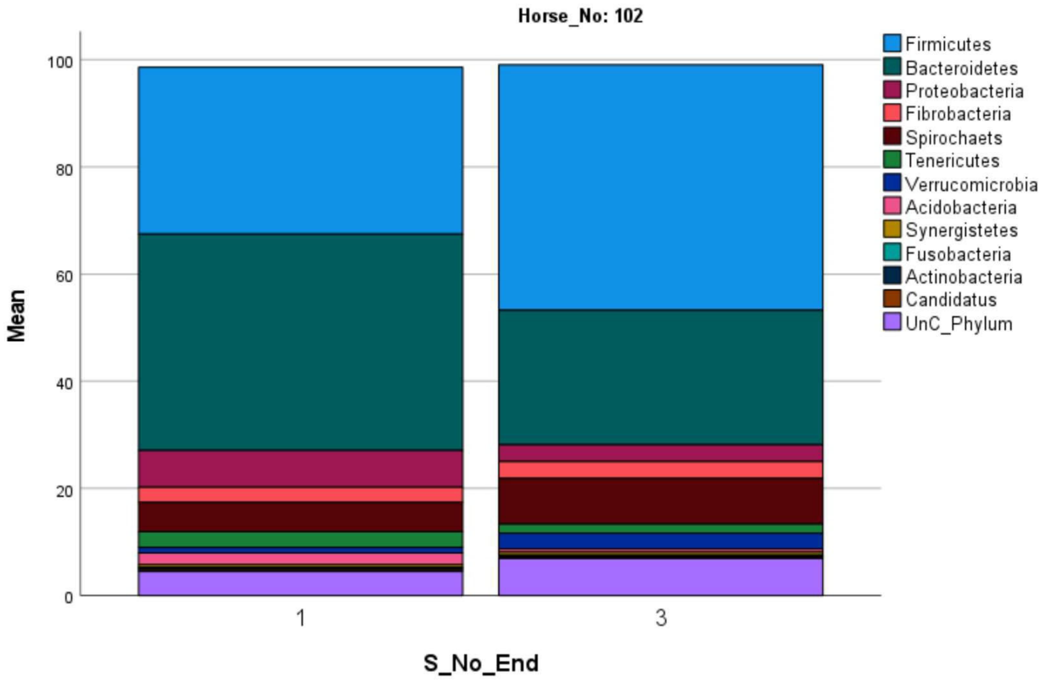

107: improvement (loose definition only) – used in intention to treat analysis only

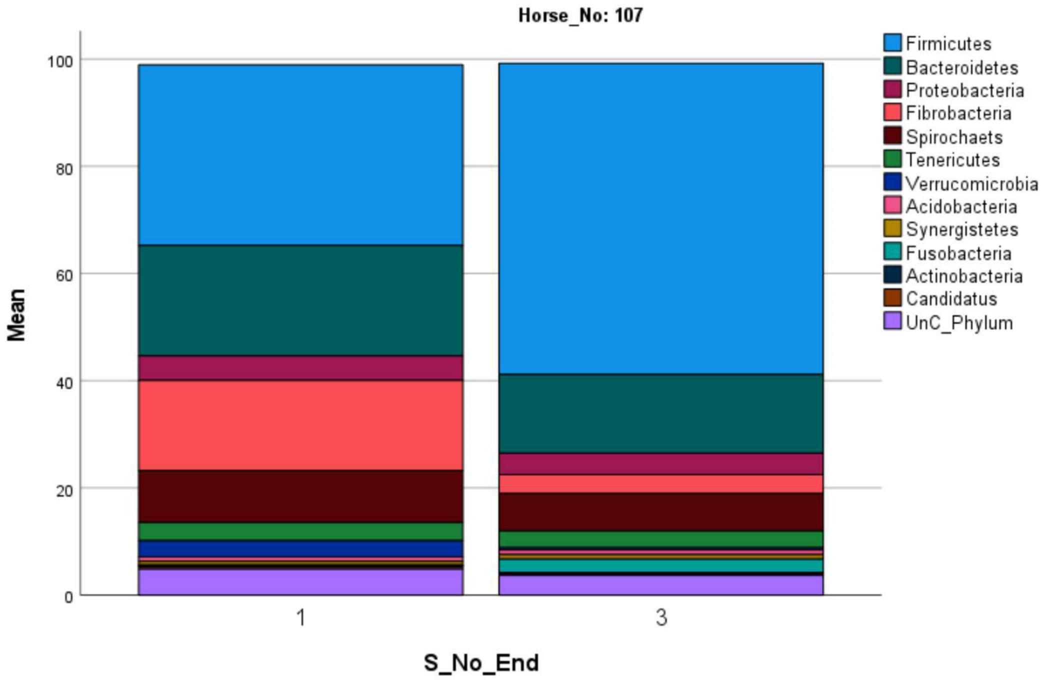

109: improvement

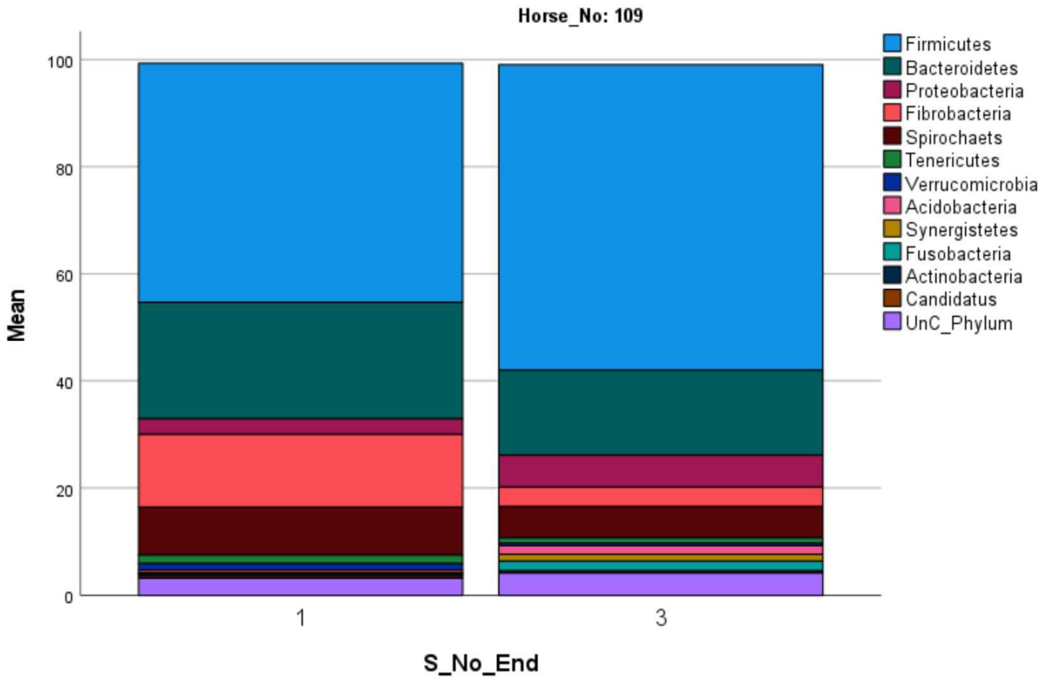

115: no improvement

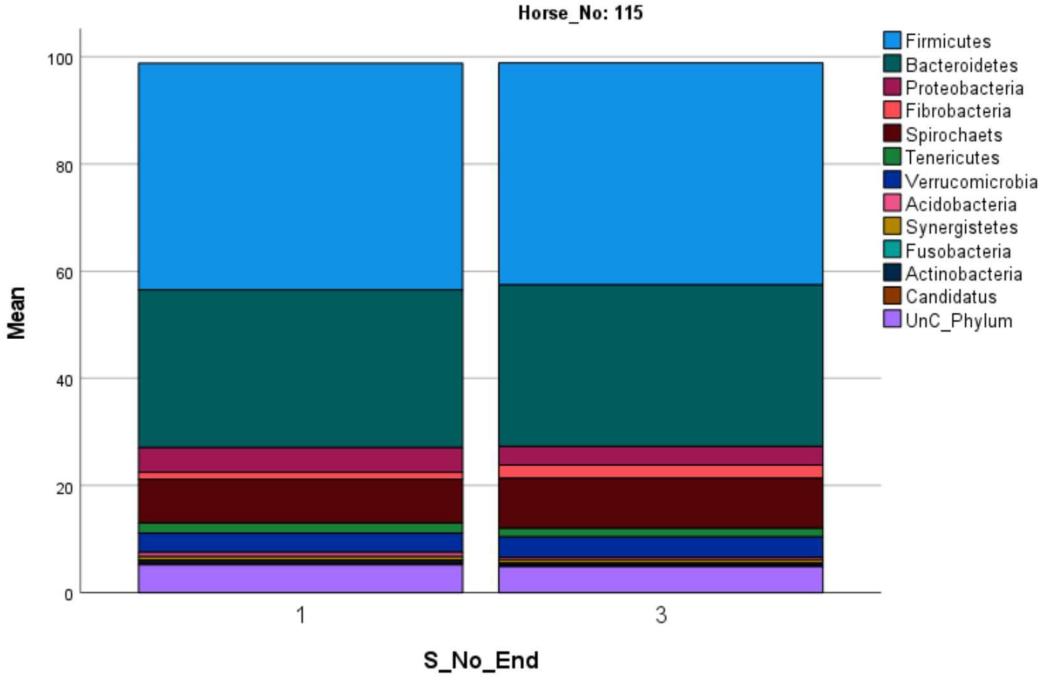

117: improvement

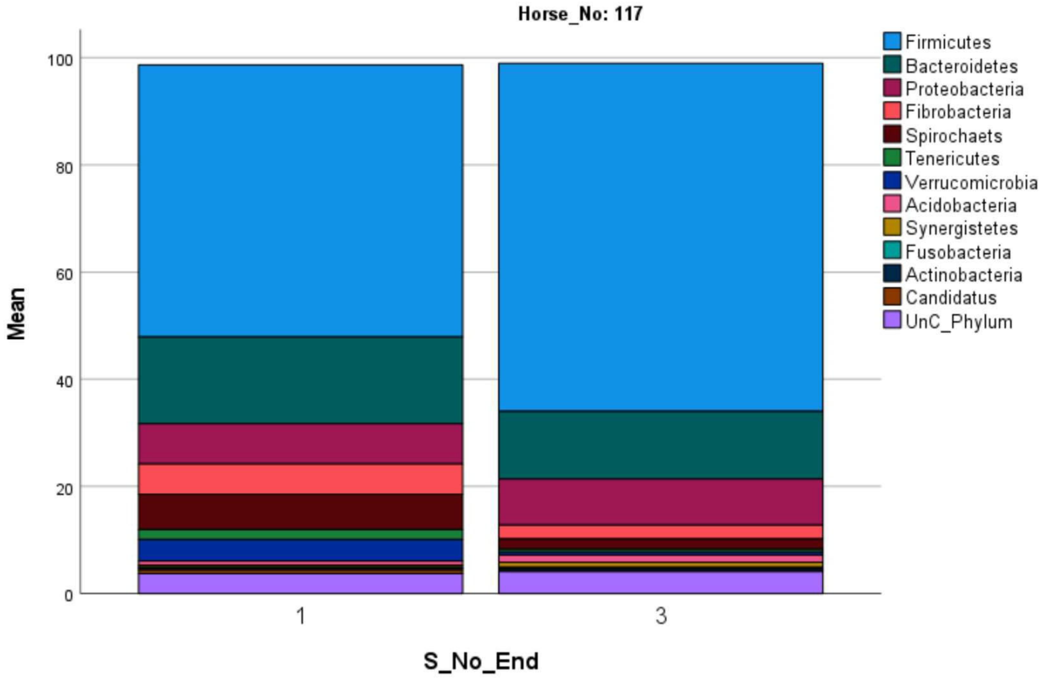

119: no improvement

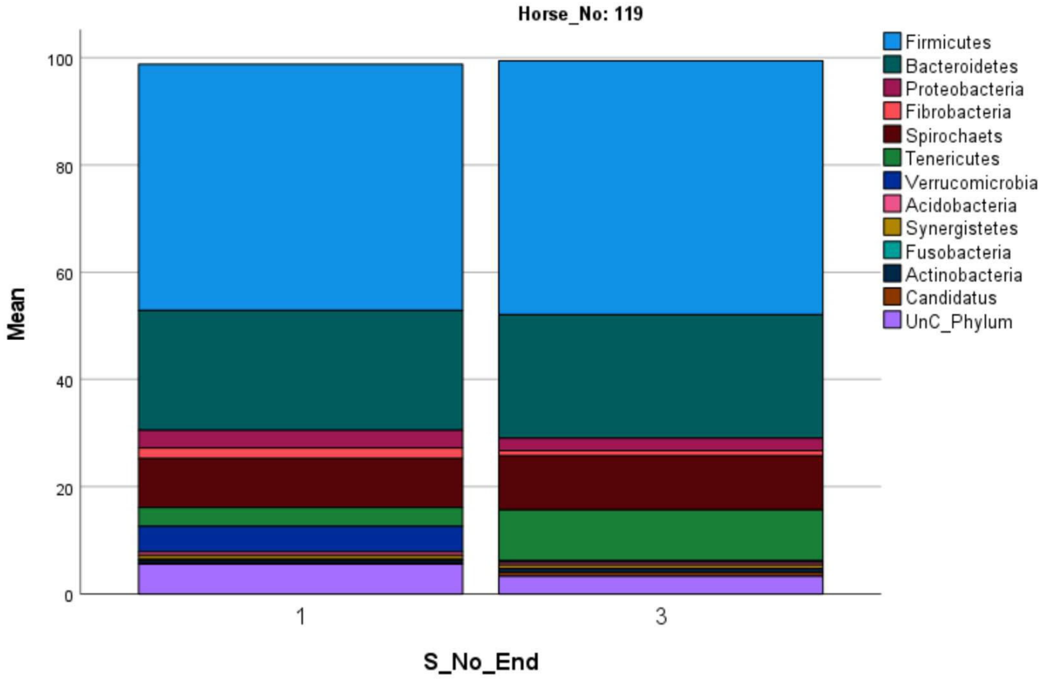

120: improvement

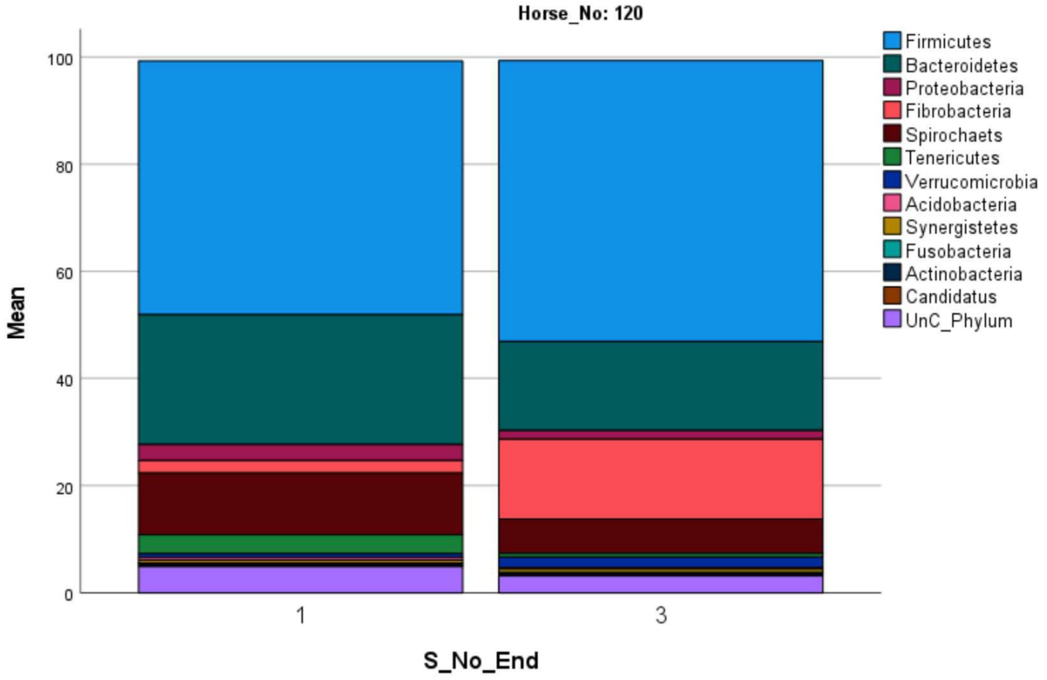

124: improvement; phenylbutazone before last sample

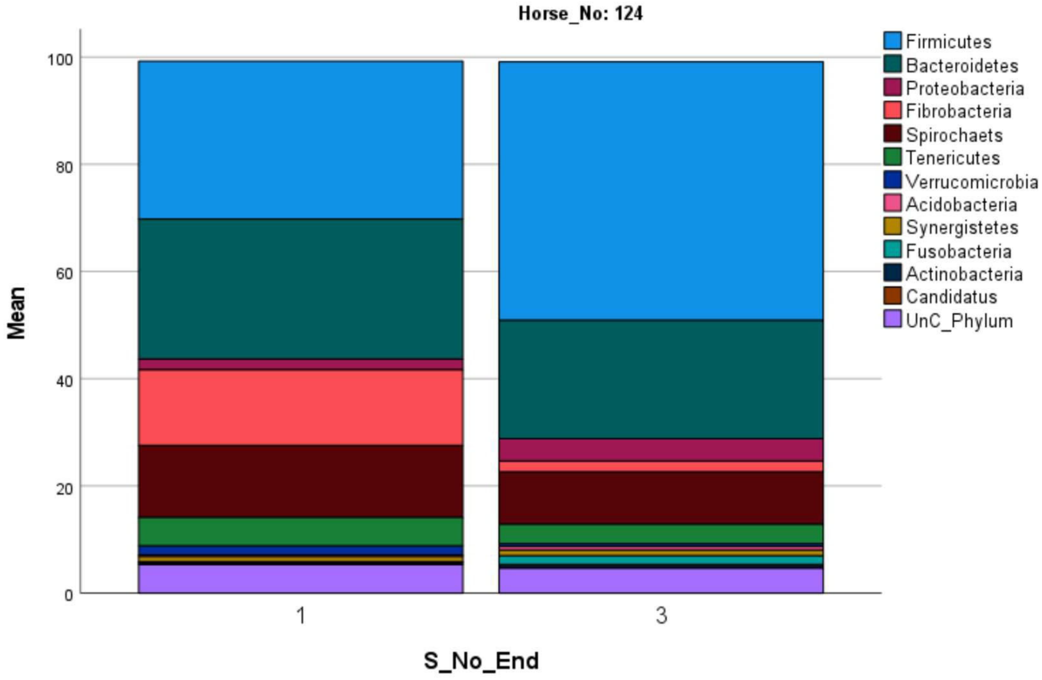

126: improvement

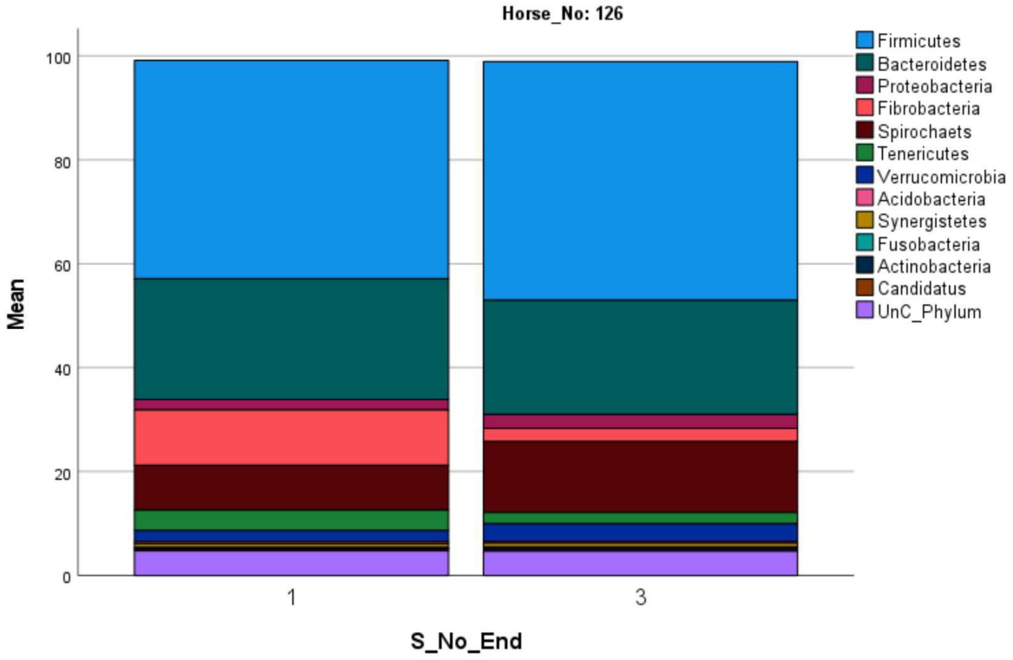

127: improvement; phenylbutazone and omeprazole before second sample

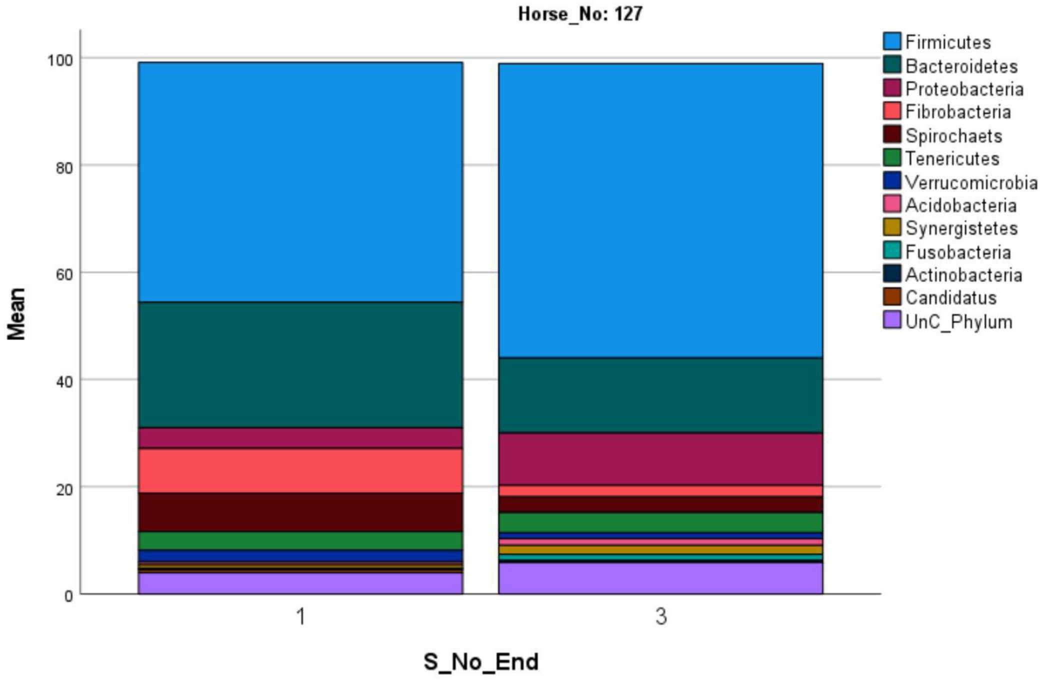

129: no improvement – used in intention to treat analysis only

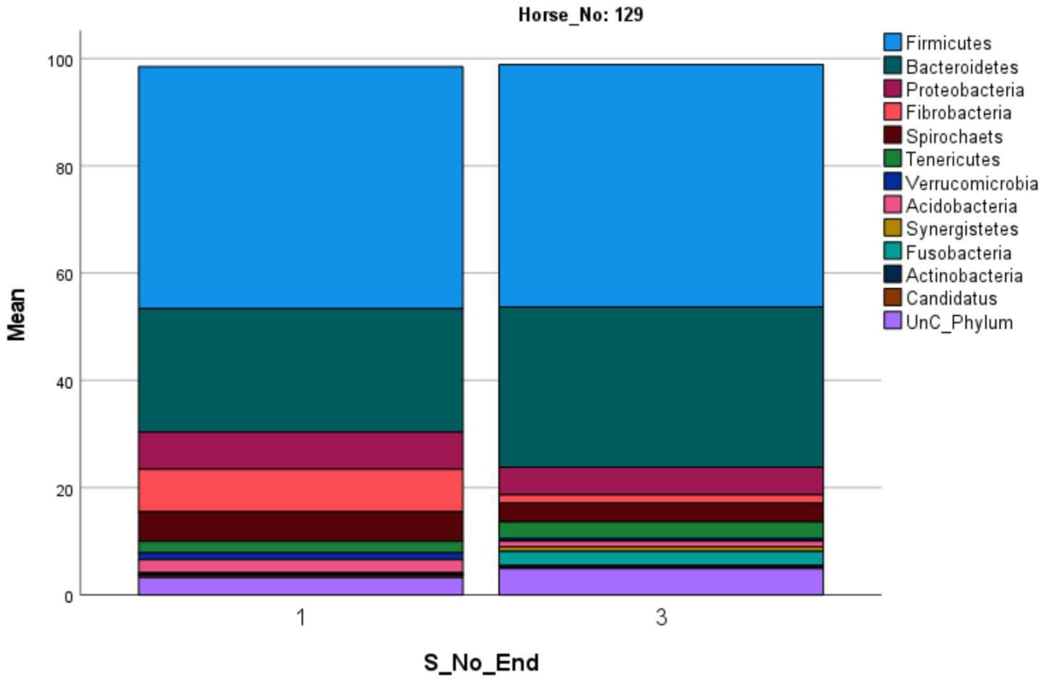

Figure S3. Stacked bar charts showing relative bacterial phyla abundances and improvement assessment by horse for 12 treated horses.

103: no improvement; omeprazole before first sample and phenylbutazone before last sample

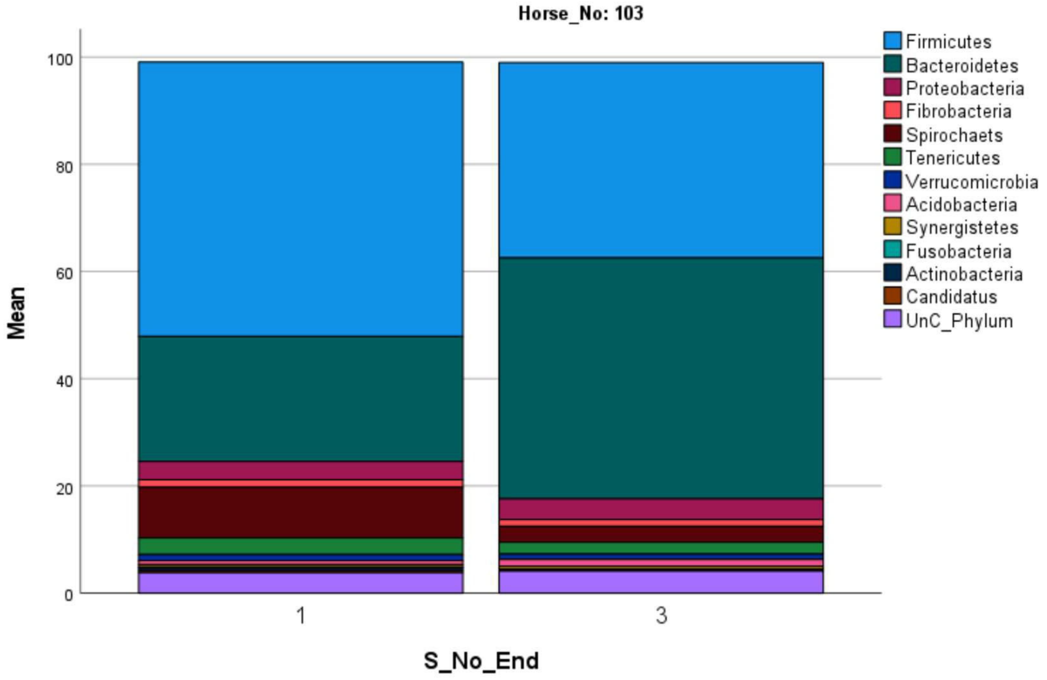

105: no improvement

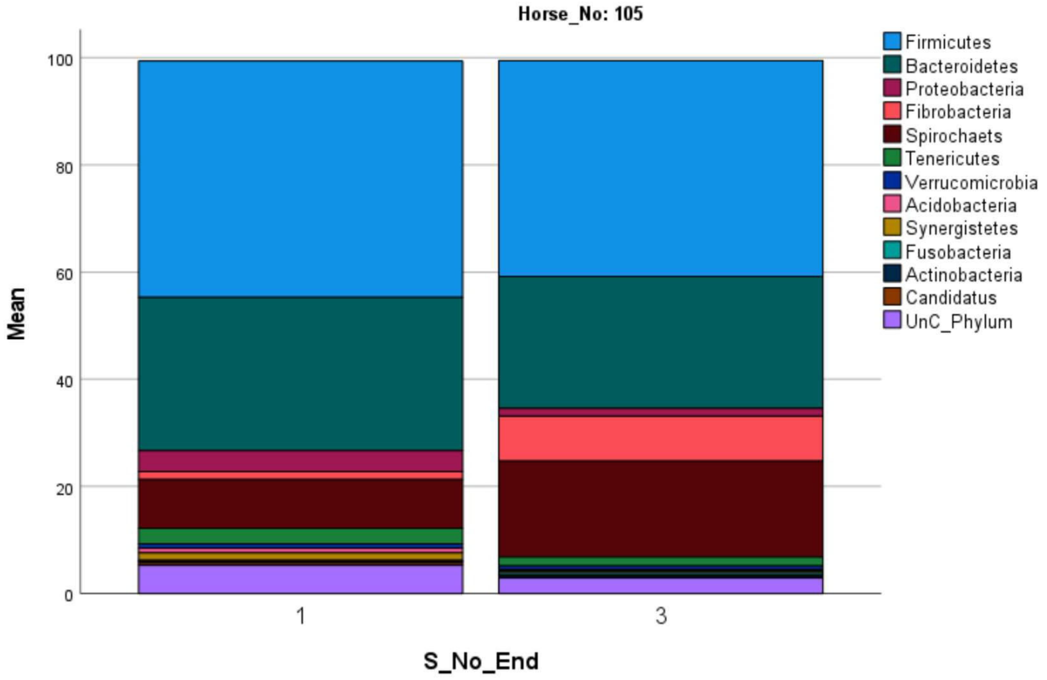

106: no improvement

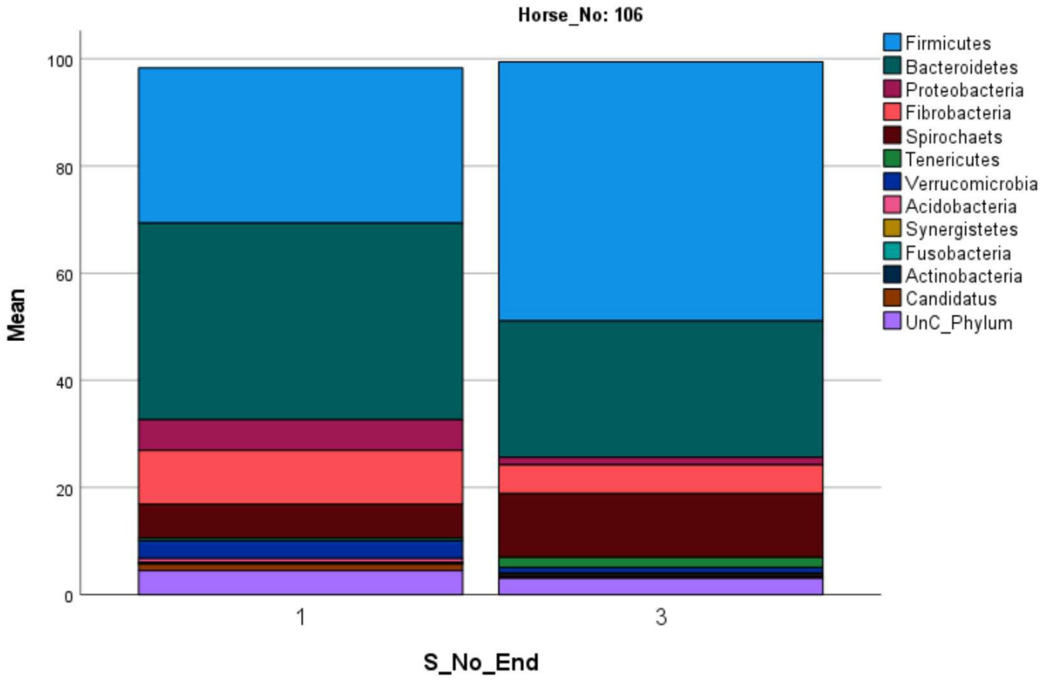

108: no improvement

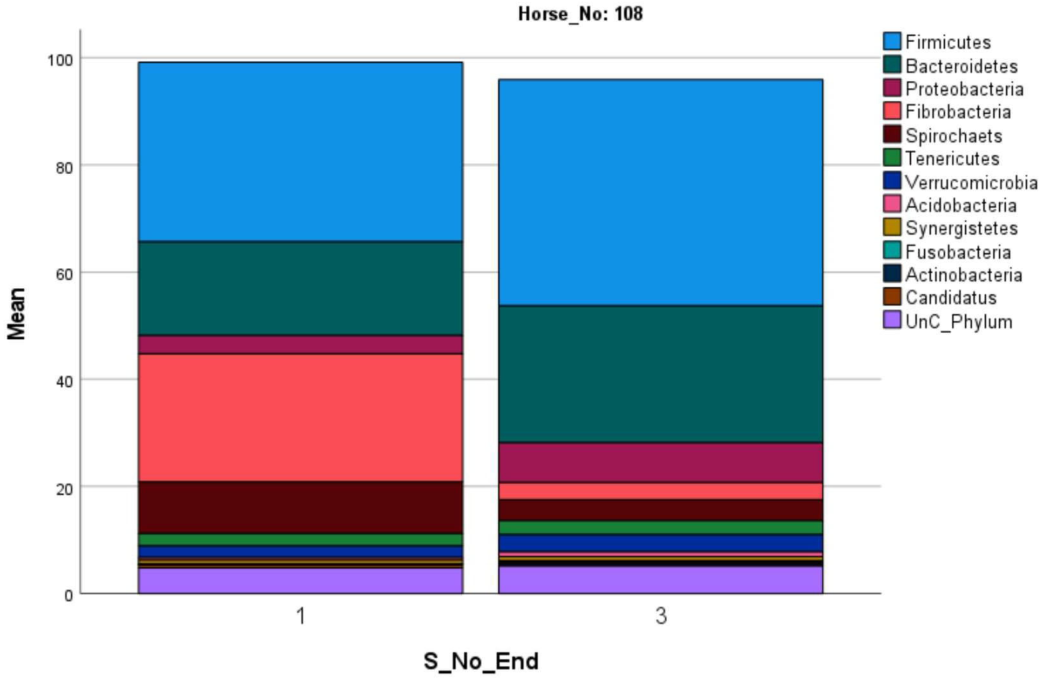

110: improvement (loose definition only)

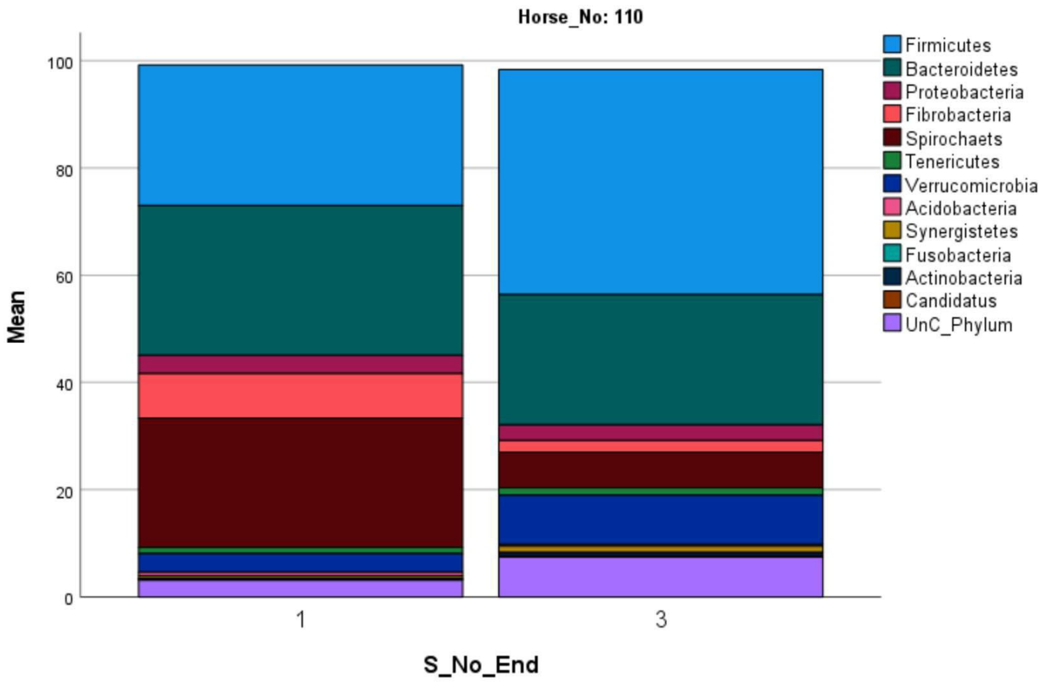

114: no improvement

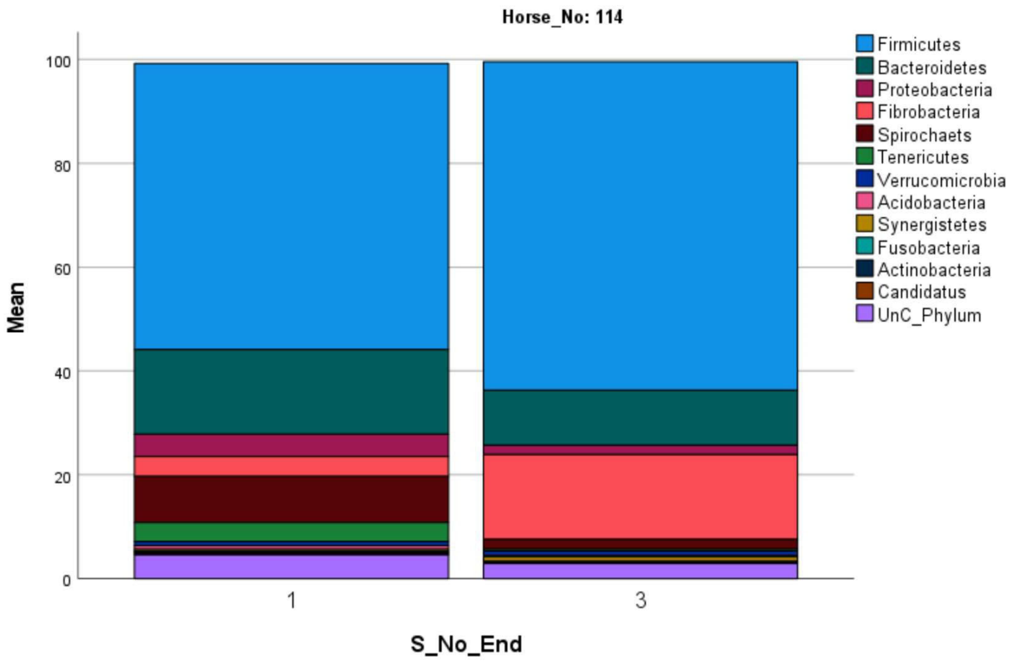

122: no improvement

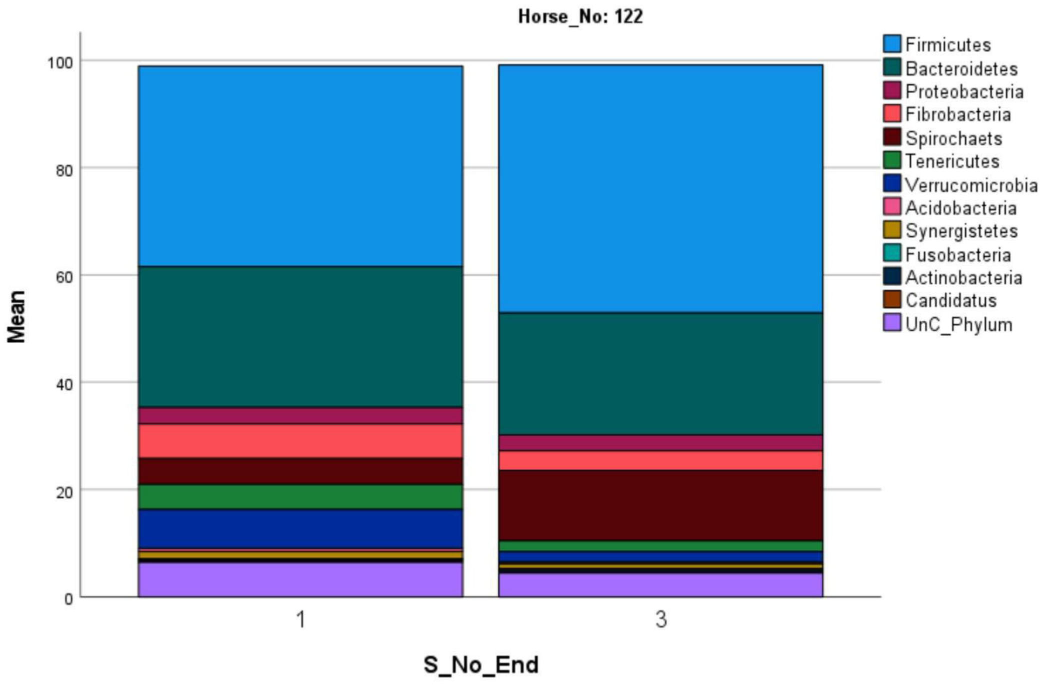

123: improvement (loose definition only)

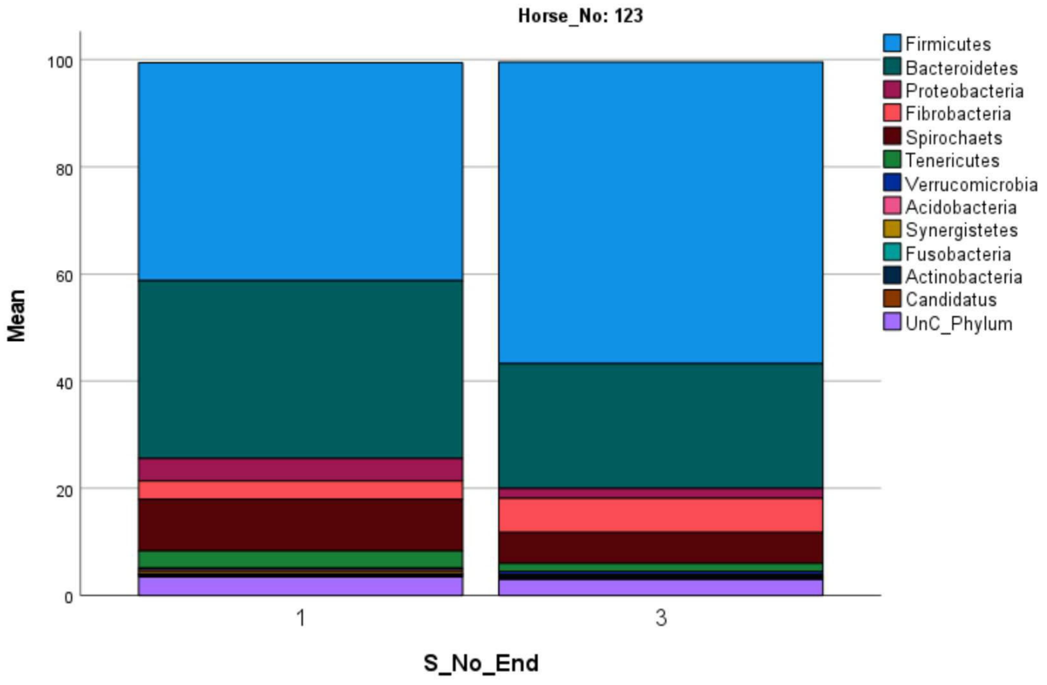

133: no improvement; phenylbutazone before last sample

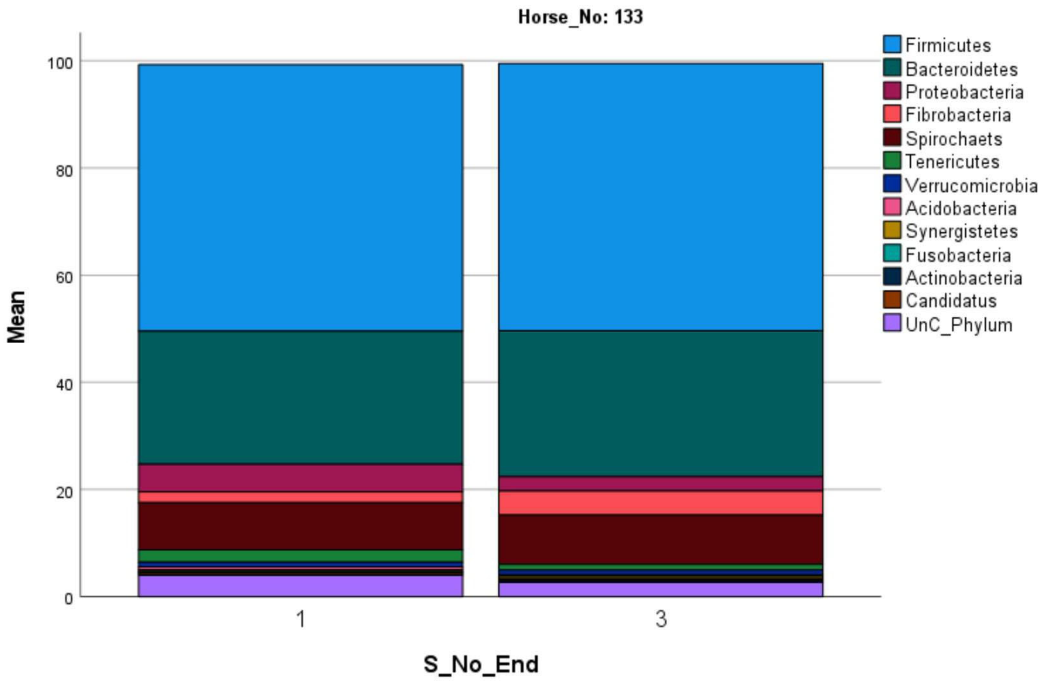

134: no improvement

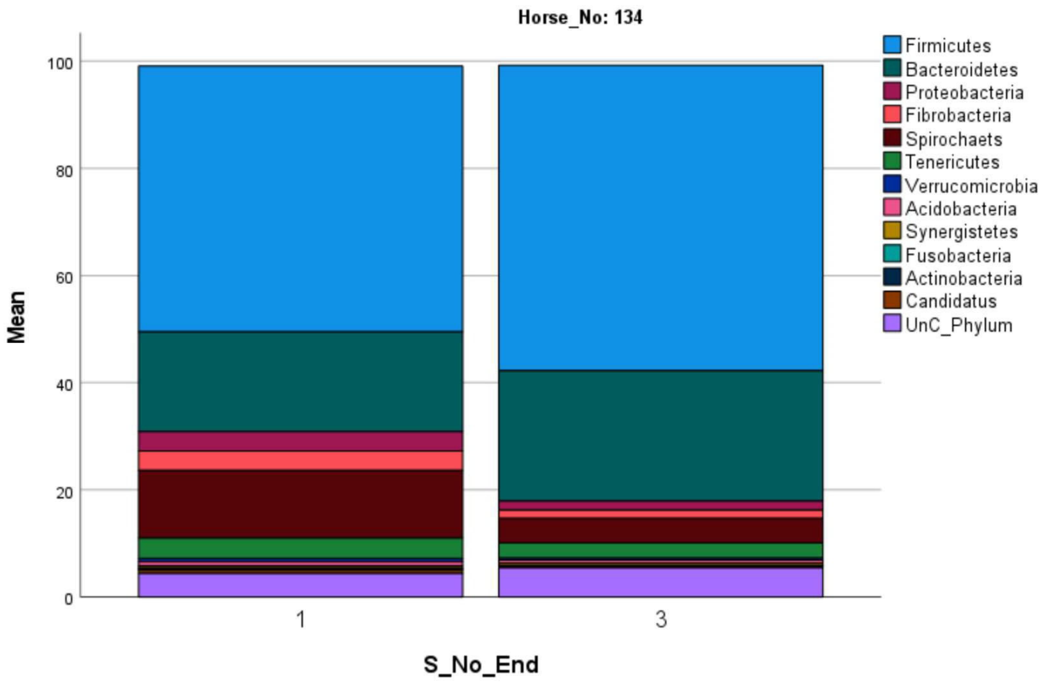

Figure S4. Stacked bar charts showing relative bacterial phyla abundances and improvement assessment by horse for 10 control horses.
